# A nomogram for predicting pathologic node negativity after neoadjuvant chemotherapy in breast cancer patients: a nationwide, multicenter retrospective cohort study (CSBrS-012)

**DOI:** 10.3389/fonc.2024.1326385

**Published:** 2024-05-10

**Authors:** Amina Maimaitiaili, Yijun Li, Na Chai, Zhenzhen Liu, Rui Ling, Yi Zhao, Hongjian Yang, Yunjiang Liu, Ke Liu, Jianguo Zhang, Dahua Mao, Zhigang Yu, Yinhua Liu, Peifen Fu, Jiandong Wang, Hongchuan Jiang, Zuowei Zhao, Xingsong Tian, Zhongwei Cao, Kejin Wu, Ailin Song, Feng Jin, Puzhao Wu, Jianjun He, Zhimin Fan, Huimin Zhang

**Affiliations:** ^1^ Department of Breast Surgery, The First Affiliated Hospital of Xi’an Jiaotong University, Xi’an, China; ^2^ Department of Breast Disease, Henan Breast Cancer Center, Affiliated Cancer Hospital of Zhengzhou University and Henan Cancer Hospital, Zhengzhou, China; ^3^ Department of Thyroid, Breast and Vascular Surgery, Xijing Hospital, Fourth Military Medical University, Xi’an, China; ^4^ Surgical Oncology Department, Shengjing Hospital of China Medical University, Shenyang, China; ^5^ Department of Breast Surgery, The Cancer Hospital of the University of Chinese Academy of Sciences (Zhejiang Cancer Hospital), Institute of Basic Medicine and Cancer (IBMC), Chinese Academy of Sciences, Hangzhou, China; ^6^ Department of Surgery, The Fourth Hospital of Hebei Medical University, Shijiazhuang, China; ^7^ Fourth Department of Breast Surgery, Jilin Cancer Hospital, Changchun, China; ^8^ Department of Breast Surgery, The Second Affiliated Hospital of Harbin Medical University, Harbin, China; ^9^ Department of Breast Surgery, Affiliated Wudang Hospital of Guizhou Medical University, Guiyang, China; ^10^ Department of Breast Surgery, The Second Hospital, Cheeloo College of Medicine, Shandong University, Jinan, China; ^11^ Breast Disease Center, Peking University First Hospital, Beijing, China; ^12^ Department of Breast Surgery, The First Affiliated Hospital, School of Medicine, Zhejiang University, Hangzhou, China; ^13^ Department of General Surgery, The First Medical Center, Chinese PLA General Hospital, Beijing, China; ^14^ Department of Breast Surgery, Beijing Chaoyang Hospital, Capital Medical University, Beijing, China; ^15^ Department of Breast Surgery, The Second Affiliated Hospital of Dalian Medical University, Dalian, China; ^16^ Department of Breast and Thyroid Surgery , Shandong Provincial Hospital Affiliated to Shandong University, Jinan, China; ^17^ Department of Thyroid, Breast, Hernia Surgery, The Inner Mongolia Autonomous Region People’s Hospital, Hohhot, China; ^18^ Department of Breast Surgery, Obstetrics and Gynecology Hospital of Fudan University, Shanghai, China; ^19^ Department of General Surgery, Lanzhou University Second Hospital, Lanzhou, China; ^20^ Department of Breast Surgery, The First Hospital of China Medical University, Shenyang, China; ^21^ Department of Vascular Surgery/Interventional Medicine, Xiang yang No.1 People’s Hospital, Hubei University of Medicine, Xiangyang, China; ^22^ Department of Breast Surgery, The First Hospital of Jilin University, Changchun, China

**Keywords:** breast cancer, neoadjuvant chemotherapy, pathologic nodal response, prediction nomogram, pathologic complete response

## Abstract

**Purpose:**

This study aimed to investigate the factors associated with pathologic node-negativity (ypN0) in patients who received neoadjuvant chemotherapy (NAC) to develop and validate an accurate prediction nomogram.

**Methods:**

The CSBrS-012 study (2010–2020) included female patients with primary breast cancer treated with NAC followed by breast and axillary surgery in 20 hospitals across China. In the present study, 7,711 eligible patients were included, comprising 6,428 patients in the primary cohort from 15 hospitals and 1,283 patients in the external validation cohort from five hospitals. The hospitals were randomly assigned. The primary cohort was randomized at a 3:1 ratio and divided into a training set and an internal validation set. Univariate and multivariate logistic regression analyses were performed on the training set, after which a nomogram was constructed and validated both internally and externally.

**Results:**

In total, 3,560 patients (46.2%) achieved ypN0, and 1,558 patients (20.3%) achieved pathologic complete response in the breast (bpCR). A nomogram was constructed based on the clinical nodal stage before NAC (cN), ER, PR, HER2, Ki67, NAC treatment cycle, and bpCR, which were independently associated with ypN0. The area under the receiver operating characteristic curve (AUC) for the training set was 0.80. The internal and external validation demonstrated good discrimination, with AUCs of 0.79 and 0.76, respectively.

**Conclusion:**

We present a real-world study based on nationwide large-sample data that can be used to effectively screen for ypN0 to provide better advice for the management of residual axillary disease in breast cancer patients undergoing NAC.

## Introduction

With the recognition of the importance of biology and systematic therapy in local control, we gradually agree that larger surgery does not cure bad biology in breast cancer (1). The adoption of a true multidisciplinary treatment approach, rather than the sequential use of different therapies, decreases the extent of surgery and its associated morbidity (2, 3).

Axillary lymph node dissection (ALND) has traditionally been used as routine axillary surgical management for breast cancer patients (4). Multiple prospective, randomized trials led by the American College of Surgeons Oncology Group (ACOSOG) Z0011 trial (5) demonstrated that sentinel lymph node biopsy (SLNB) can replace ALND in patients with low nodal burden disease because of noninferior local control and survival, but with lower surgical morbidity. Neoadjuvant chemotherapy (NAC) results in frequent downstaging of tumors in both the breast and axilla, which can lead to fewer surgeries in patients with larger tumors at diagnosis. The implementation of NAC has enabled selected women to undergo breast-conserving surgery (BCS) in the last two decades; however, for patients who received NAC, the chance of de-escalated axillary surgery has not improved (6). The National Comprehensive Cancer Network (NCCN) breast cancer guidelines recommend SLNB for patients with cN0 to ycN0 disease after NAC, but ALND is still recommended for patients who are converted from cN+ to cN0, and SLNB is usually considered a relative contraindication due to its low identification rate and high false-negative rate (FNR) (7, 8). In the SENTinel NeoAdjuvant (SENTINA) study (9), the detection rate of SLNB after NAC in patients with cN+ to cN0 disease was 80.1% (95% CI 76.6–83.2), and the false-negative rate was 14.2% (95% CI 9.9–19.4). However, approximately 74% of breast cancer patients with cN0 disease are sentinel lymph node-negative, and postoperative complications still occur even after SLNB (10, 11).

Patients with a low risk of residual axillary involvement after NAC could benefit from omitting axillary surgical intervention if there are accurate tools for nodal response prediction (12). Currently, the commonly used clinical imaging methods for evaluating the axillary region include ultrasound, mammography, magnetic resonance imaging (MRI), and positron emission tomography CT (PET-CT) (13–15). Nevertheless, the accuracy of these techniques remains low, and there are no unified guidelines for axillary imaging evaluation of NAC response (16). The ACOSOG Z1071 (Alliance) trial reported that axillary ultrasound (AUS) after NAC can identify abnormal nodes, guide patient selection for SLN surgery instead of ALND, and reduce the FNR of SLNB to less than 10%. However, the accuracy of AUS after NAC was low; only 43.2% of patients who were negative for AUS were confirmed to have nodal pCR by ALND (16). Investigators also attempted to decrease the FNR by marking positive lymph nodes at diagnosis before NAC. However, clips can be found during surgery in only half of the patients (17). Imaging-guided localization (IGL) of the clipped node was introduced to increase the likelihood of clip removal. The lowest FNR was achieved when IGL was added to SLN biopsy, a procedure called targeted axillary dissection (TAD) (18, 19). However, it did not significantly change the performance of tailored axillary surgery, which left ≥2 positive nodes behind in 47.6% of the patients (20, 21). In all these explorations, the prediction model based on clinical and pathological factors still has clinical value and application prospects. The present study aimed to identify factors that are predictive of ypN0 and construct a novel nomogram that can effectively predict nodal negativity and thus potentially avoid axillary surgery, which can reduce women’s loss of function and lymphedema.

## Methods

### Study population

**Figure 1:**
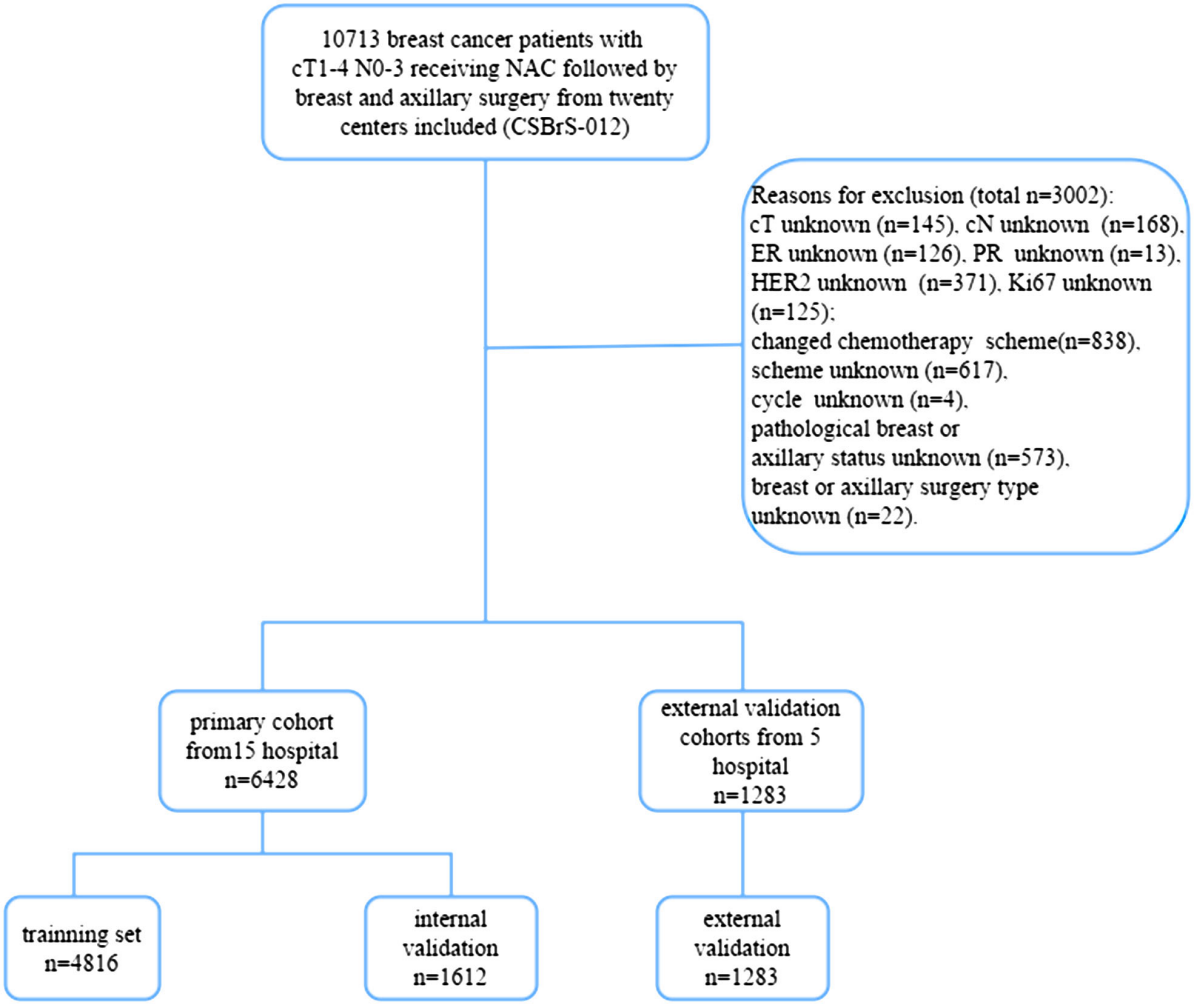
Flow diagram of the study. NAC, neoadjuvant chemotherapy; CSBrS-012: The Chinese Society of Breast Surgery study (2010–2020).

The Chinese Society of Breast Surgery (CSBrS-012) is a nationwide, multicenter, 10-year retrospective clinical epidemiological study conducted across 20 hospitals in China. The CSBrS-012 study included female primary breast cancer patients who received NAC and underwent standard breast and axillary surgery after NAC between January 2010 and December 2020. The 20 hospitals are located in central, northern, eastern, northwestern, northeastern, and southwestern China, and represent different levels of breast cancer burden. After excluding patients with incomplete data, 7,711 patients were enrolled in the study. Hospitals were randomly assigned to two groups comprising 6,428 patients in the primary cohort from 15 hospitals and 1,283 patients in the external validation cohort from five hospitals. We then randomized the patients in the primary cohort at a 3:1 ratio into the training and internal validation sets (**Figure 1**). The study was performed in accordance with the Declaration of Helsinki and approved by the Ethical Review Committee of the First Hospital of Jilin University (No. 2021–066). As this was a retrospective study and all data analyses were performed anonymously, the requirement for informed consent from the patients was waived.

### Patient characteristics

Variables included age, clinical tumor (cT) and clinical nodal (cN) stages before NAC, tumor histology, ER, PR, HR, HER2, Ki-67, biological subtypes, NAC regimen, NAC treatment cycle, and pCR status. Immunohistochemistry (IHC) was used to detect the expression of ER, PR, HER2, and Ki-67. ER and PR were defined as positive if ≥1% of cells were positive. HR was defined as positive if the ER and/or PR were positive. HER2 expression was defined as positive if 3+ by IHC or 2+ by IHC and positive by *in situ* hybridization. Tumor subtypes were categorized according to St. Gallen criteria (22): HR+/HER2−, HR+/HER2+, HR−/HER2+, and TNBC. The T and N stages were defined according to the 8th edition of the American Joint Committee on Cancer (AJCC) (23). cN0 was defined as no suspicious lymph nodes on axillary ultrasound or suspicious lymph nodes on axillary ultrasound but negative on either fine needle aspiration cytology or core needle biopsy or negative on SLNB prior to NAC. Suspicious lymph nodes were considered in cases of a hypoechoic round shape, focally thickened cortex, or absent fatty hilum. pCR was defined as the absence of residual invasive or *in situ* carcinoma in the breast or axillary lymph nodes (ypT0/ypN0). NAC and surgery were performed in accordance with the Chinese Society of Clinical Oncology (CSCO) Breast Cancer Guidelines and the National Comprehensive Cancer Network (NCCN) Breast Cancer Guidelines. In our study, we divided NAC regimens into three categories: (1) anthracycline combined with taxane, (2) taxane combined with platinum, and (3) other regimens.

### Statistical analysis

Statistical analyses were performed using SPSS version 21.0 (Inc., Chicago, IL, USA) and R 4.2.2 (R Project for Statistical Computing) software. The differences in clinicopathological parameters between the training and internal validation sets were evaluated using Pearson’s *χ* ^2^ test. Univariate logistic regression and backward stepwise selection were used for the final multivariate model. A predictive nomogram for ypN0 was established based on independent risk factors identified via multivariate analysis. The predictive value of the model was appraised using receiver operating characteristic (ROC) curves and calibration curves. The AUC (area under the receiver operating characteristic curve) was calculated.

## Results

### Patient characteristics and NAC response

A total of 7,711 female breast cancer patients, with a median age of 49 years, were enrolled. The baseline characteristics of the patients are summarized in **Table 1**. The proportion of patients with initial stage cT1–2 tumors was 79.8%, and that with initial stage cT3–4 tumors was 20.2%. The proportion of patients with cN0–1 stage disease in the study population was greater than that of patients with cN2–3 stage disease (81.5% vs. 18.5%). Most patients had invasive ductal cancer (6,971 [90.4%]). Anthracyclines and taxanes were the most common NAC treatments (76.3%). Approximately half of the HER2+patients received targeted therapy, among which single-agent HER2 blockade was more than twice as frequent as dual HER2 blockade.

**Table 1:** Baseline patient characteristics.

Characteristic	Training set (n = 4,816)	Internal validation set (n = 1,612)	p-value	External Validation set (n = 1,283)
Age			0.155	
≤35	482 (10.0)	181 (11.2)		134 (10.4)
≥56	1,216 (25.2)	379 (23.5)		374 (29.2)
36–45	1,265 (26.3)	401 (24.9)		320 (24.9)
46–55	1,853 (38.5)	651 (40.4)		455 (35.5)
cT			0.712	
T1	583 (12.1)	174 (10.8)		205 (16.0)
T2	3,234 (67.2)	1,096 (68.0)		859 (67.0)
T3	749 (15.5)	256 (15.9)		173 (13.4)
T4	250 (5.2)	86 (5.3)		46 (3.6)
cN			0.551	
N0	1,390 (28.9)	450 (28.0)		318 (24.7)
N1	2,590 (53.8)	871 (54.0)		666 (52.0)
N2	338 (7.0)	174 (10.8)		210 (16.4)
N3	498 (10.3)	117 (7.2)		89 (6.9)
Histology			0.032	
IDC	4,306 (89.4)	1,472 (91.3)		1,193 (93.0)
Others	510 (10.6)	140 (8.7)		90 (7.0)
Biologic Subtype			0.034	
HR−/HER2−	858 (17.8)	239 (14.8)		241 (18.8)
HR−/HER2+	703 (14.6)	232 (14.4)		160 (12.5)
HR+/HER2−	2,290 (47.5)	815 (50.6)		626 (48.8)
HR+/HER2+	965 (20.0)	326 (20.2)		256 (20.0)
HER2			0.308	
Negative	3,148 (65.4)	1,054 (65.4)		867 (67.6)
Positive no target	873 (18.1)	301 (18.7)		163 (12.7)
Positive + single agent HER2 blockade	590 (12.3)	176 (10.9)		165 (12.9)
Positive + dual HER2 blockade	205 (4.3)	81 (5.0)		88 (6.9)
Ki67			0.497	
<20%	682 (14.2)	240 (14.9)		193 (15.0)
≥20%	4,134 (85.8)	1,372 (85.1)		1,090 (85.0)
NAC regimen			0.873	
Anthracyclines + Taxanes	3,758 (78.0)	1,263 (78.3)		860 (67.0)
Taxanes + Platinums	448 (9.3)	143 (8.9)		187 (14.6)
Others	610 (12.7)	206 (12.8)		236 (18.4)
Cycle			0.405	
4	580 (12.0)	219 (13.6)		377 (29.4)
6	1,936 (40.2)	656 (40.7)		507 (39.5)
8	1,592 (33.1)	521 (32.3)		287 (22.4)
>8	306 (6.4)	93 (5.8)		24 (1.9)
Others	402 (8.3)	123 (7.6)		88 (6.9)
bpCR			0.061	
No	3,801 (78.9)	1,308 (81.1)		1,044 (81.4)
Yes	1,015 (21.1)	304 (18.9)		239 (18.6)

cT, clinical tumor stage before neoadjuvant chemotherapy; cN, clinical tumor stage before neoadjuvant chemotherapy; IDC, invasive ductal carcinoma; ER, estrogen receptor; PR, progesterone receptor; HR, hormone receptor; HER2, human epidermal growth factor receptor 2; bpCR, breast pathologic complete response.

As shown in **Table 2**, 3,560 patients (46.2%) achieved ypN0, and 1,558 patients (20.3%) achieved bpCR. Among the patients who achieved bpCR, 75.3% had ypN0, whereas 38.7% did not (*p <*0.001). The pathological responses of the breast and axillary lymph nodes according to the biological subtype are summarized in **Table 3**. Responses to NAC in the different subgroups were generally consistent between the breast and axillary regions. In both the breast and axilla, HR-negative patients showed a better response to NAC than HR-positive patients (*p <*0.001). In both the breast and axilla, HR+/HER2− subtypes exhibited relatively poor responses to NAC compared to the other subtypes (*p <*0.001). The ypN0 rate for all subtypes was significantly higher than the bpCR rate (*p <*0.05).

**Table 2:** Pathologic response of breast and axillary to NAC in whole study population.

Response	ypN0	ypN+	total
bpCR	1,173 (75.3)	385 (24.7)	1,558 (20.3)
non-bpCR	2,387 (38.7)	3,766 (61.3)	6,153 (79.7)
total	3,560 (46.2)	4,151 (53.8)	7,711

ypN0, pathologic node negative after neoadjuvant chemotherapy; ypN+, pathologic node positive after neoadjuvant chemotherapy; bpCR, breast pathologic complete response; non-bpCR, not achieved breast pathologic complete response.

**Table 3:** Pathological response of breast and axillary lymph node according to biologic subtype.

Subtypes	ypN0	ypN+	bpCR	non-bpCR	total
HR−/HER2−	793 (59.3)	545	422 (31.6)	916	1,338
HR−/HER2+	657 (60.0)	438	350 (32.0)	745	1,095
HR+/HER2−	1,268 (34.0)	2,463	437 (11.8)	3294	3,731
HR+/HER2+	842 (54.5)	705	349 (22.6)	1,198	1,547

HR, hormone receptor; HER2, human epidermal growth factor receptor; ypN0, pathologic node negative after neoadjuvant chemotherapy; ypN+, pathologic node positive after neoadjuvant chemotherapy; bpCR, breast pathologic complete response; non-bpCR, not achieved breast pathologic complete response.

### Associations between ypN0 and clinicopathologic parameters

According to the univariate logistic regression analyses of the training set, cN stage, ER, PR, HER2, Ki67, NAC treatment cycle, and bpCR were associated with ypN0. All of the above parameters were subjected to multivariate logistic regression using backward selection analysis, and a lower cN stage, ER-negative status, PR-negative status, HER2-positive status with targeted therapy, Ki67 level ≥20, more NAC treatment cycles, and bpCR were confirmed to be independent predictors of ypN0 (**Table 4**).

**Table 4:** Univariate and multivariate logistic analysis of factors predict the lymph node positivity after NAC in the training set.

Variables	Univariate Analysis			Multivariable Analysis		
	OR	95% CI	P-value	OR	95% CI	P-value
Age						
≤35	reference					
46–55	1.01	0.80–1.28	0.934			
36–45	1.16	0.91–1.49	0.231			
≥56	0.91	0.71–1.18	0.512			
cT						
T1	reference					
T2	1.05	0.86–1.30	0.619			
T3	1.02	0.79–1.33	0.830			
T4	0.77	0.54–1.10	0.152			
cN						
N0	reference			reference		
N1	0.18	0.16–0.22	<0.001	0.19	0.16–0.22	<0.001
N2	0.11	0.08–0.15	<0.001	0.11	0.08–0.14	<0.001
N3	0.09	0.07–0.12	<0.001	0.09	0.07–0.12	<0.001
Tumor Histology						
IDC	reference					
Others	1.13	0.91–1.41	0.281			
ER						
Negative	reference					
Positive	0.63	0.52–0.76	<0.001	0.62	0.51–0.75	<0.001
PR						
Negative	reference					
Positive	0.77	0.64–0.93	0.006	0.78	0.65–0.94	0.010
HER2						
Negative	reference					
Positive no target	1.44	1.21–1.72	<0.001	1.42	1.19–1.68	<0.001
Positive + single agent HER2 blockade	2.39	1.80–3.17	<0.001	2.40	1.94–2.97	<0.001
Positive + dual HER2 blockade	2.28	1.51–3.45	<0.001	2.32	1.64–3.32	<0.001
Ki67						
<20%	reference			reference		
≥20%	1.45	1.19–1.78	<0.001	1.46	1.20–1.78	<0.001
NAC regimen						
Anthracyclines + Taxanes	reference					
Taxanes + Platinums	1.02	0.71–1.46	0.914			
Others	1.00	0.81–1.23	0.983			
NAC cycle						
4	reference					
6	1.20	0.96–1.50	0.107	1.19	0.96–1.48	0.117
8	1.26	1.01–1.60	0.044	1.25	1.00–1.56	0.052
>8	1.97	1.42–2.77	<0.001	1.95	1.40–2.71	<0.001
Others	1.24	0.92–1.67	0.156	1.24	0.92–1.66	0.161
bpCR						
No	reference			reference		
Yes	4.62	3.86–5.53	<0.001	4.65	3.90–5.57	<0.001

Only variables with P-values <0.05 were included in the multivariate analysis. OR, odds ratio; 95% CI, 95% confidence interval; cT, clinical tumor stage before neoadjuvant chemotherapy; cN, clinical tumor stage before neoadjuvant chemotherapy; IDC, invasive ductal carcinoma; ER, estrogen receptor; PR, progesterone receptor; HR, hormone receptor; HER2, human epidermal growth factor receptor 2; bpCR, breast pathologic complete response.

### Nomogram for predicting ypN0

**Figure 2:**
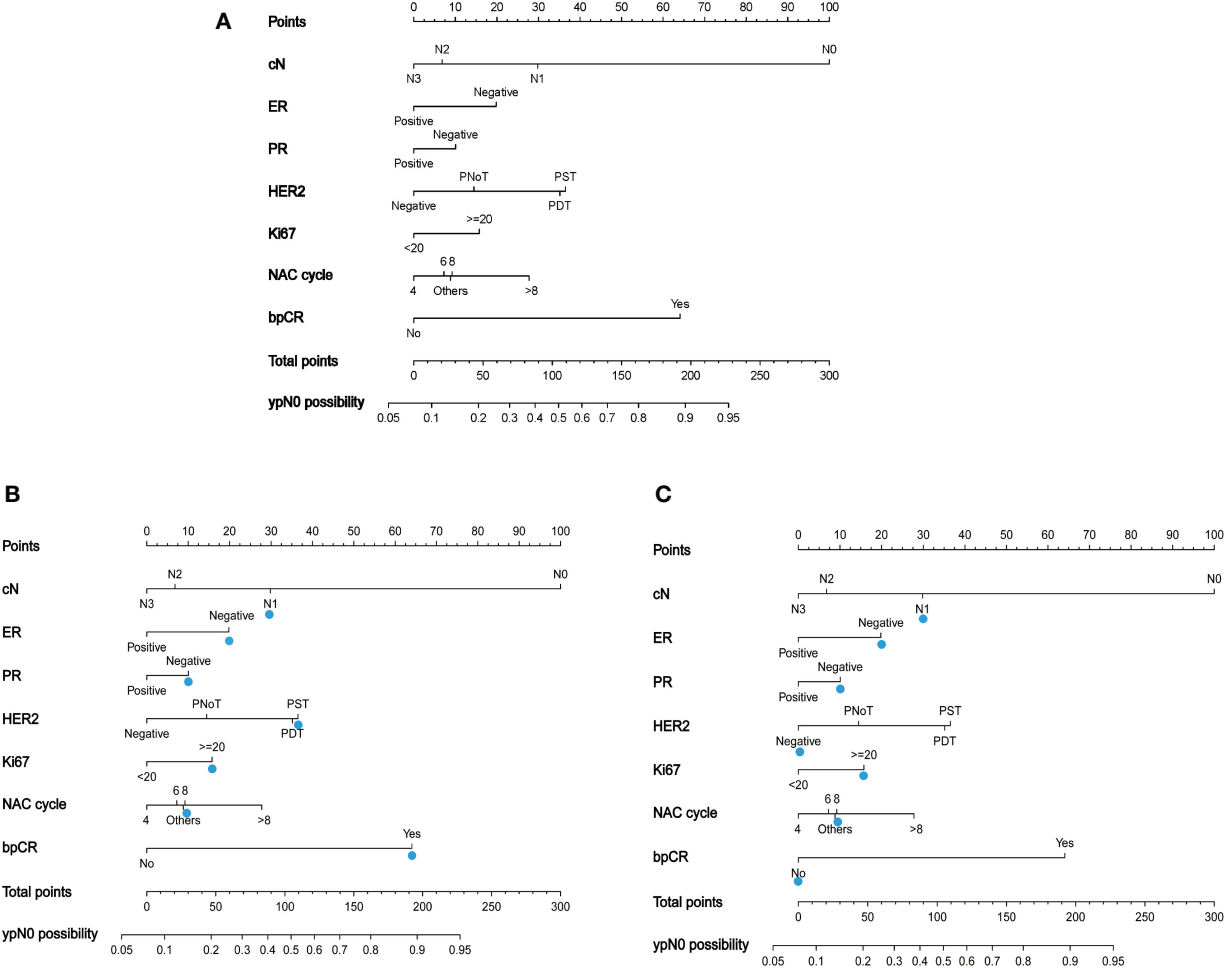
**(A)** A nomogram to predict the probability of ypN0 in breast cancer patients receiving neoadjuvant chemotherapy. PNoT, HER2 positive without targeted therapy; PST, HER2 positive with single agent HER2 blockade; PDT, HER2 positive with dual HER2 blockade. **(B)** The blue triangle demonstrates usage of the model: a patient with HER2-amplified breast cancer with cN1 and Ki67 >20 who received eight cycles of NAC with single-targeted therapy and achieved bpCR had a total of 188 points, and the possibility of ypN0 after NAC for this patient was 88%; **(C)** a patient with triple-negative breast cancer with cN1 and Ki67 >20 who received eight cycles of NAC and did not achieve bpCR had a total of 88 points. The possibility of ypN0 after NAC was 40% for this patient.

A nomogram to predict ypN0 was developed based on multivariate logistic regression results. Points were assigned to each variable and summed to obtain the total number of points. Finally, the probability of ypN0 was determined by drawing a vertical line from the total score to the bottom row (**Figure 2A**). For example, a patient with HER2-amplified breast cancer with cN1 and Ki67 >20 who received eight cycles of NAC with single targeted therapy and achieved bpCR had a total of 188 points, so the possibility of ypN0 after NAC for this patient was 88% (**Figure 2B**), and a patient with triple-negative breast cancer with cN1 and Ki67 >20 who received eight cycles of NAC and did not achieve bpCR had a total of 88 points, so the possibility of ypN0 after NAC for this patient was 40% (**Figure 2C**).

**Figure 3:**
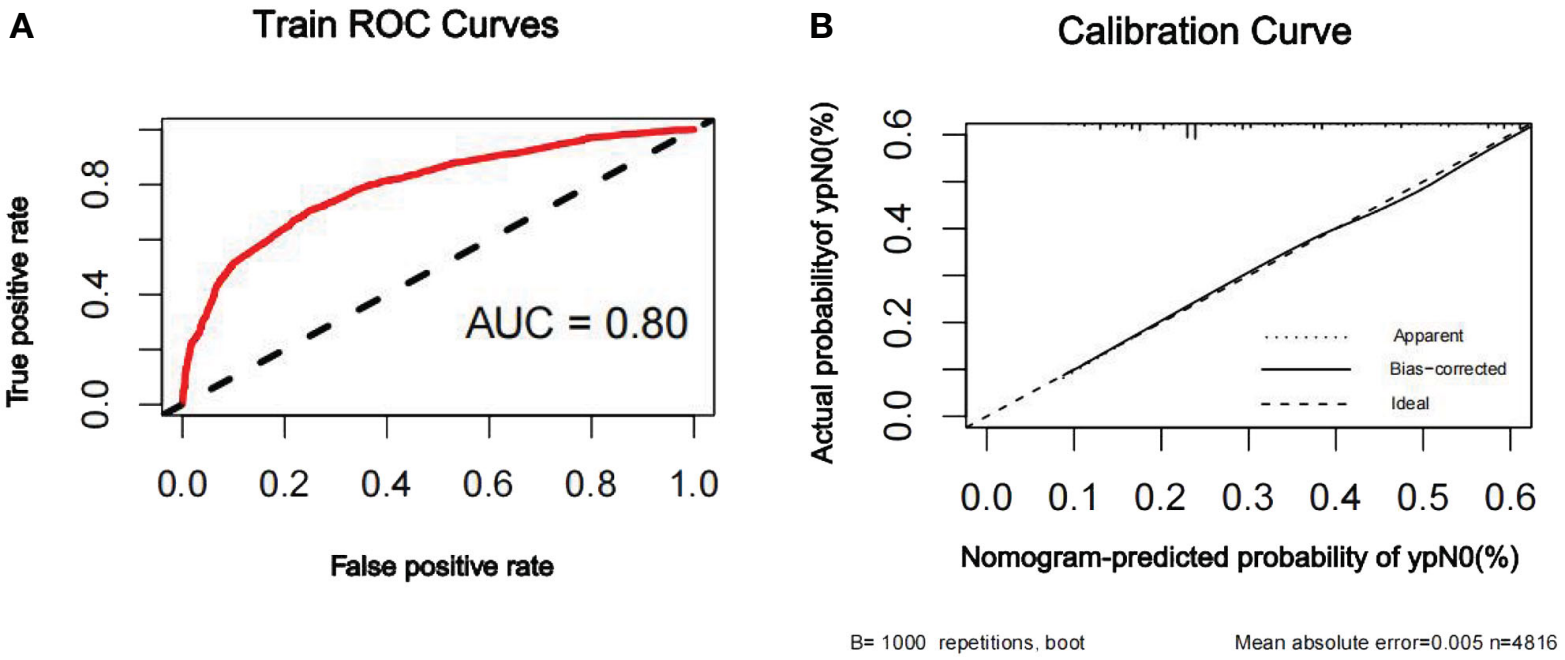
ROC curve **(A)** and calibration curve **(B)** are shown for the prediction model of ypN0 in the training cohort. The ROC curve for the training set indicated an AUC of 0.80. ROC, receiver operating characteristic curve; AUC, area under the curve.

**Figure 4:**
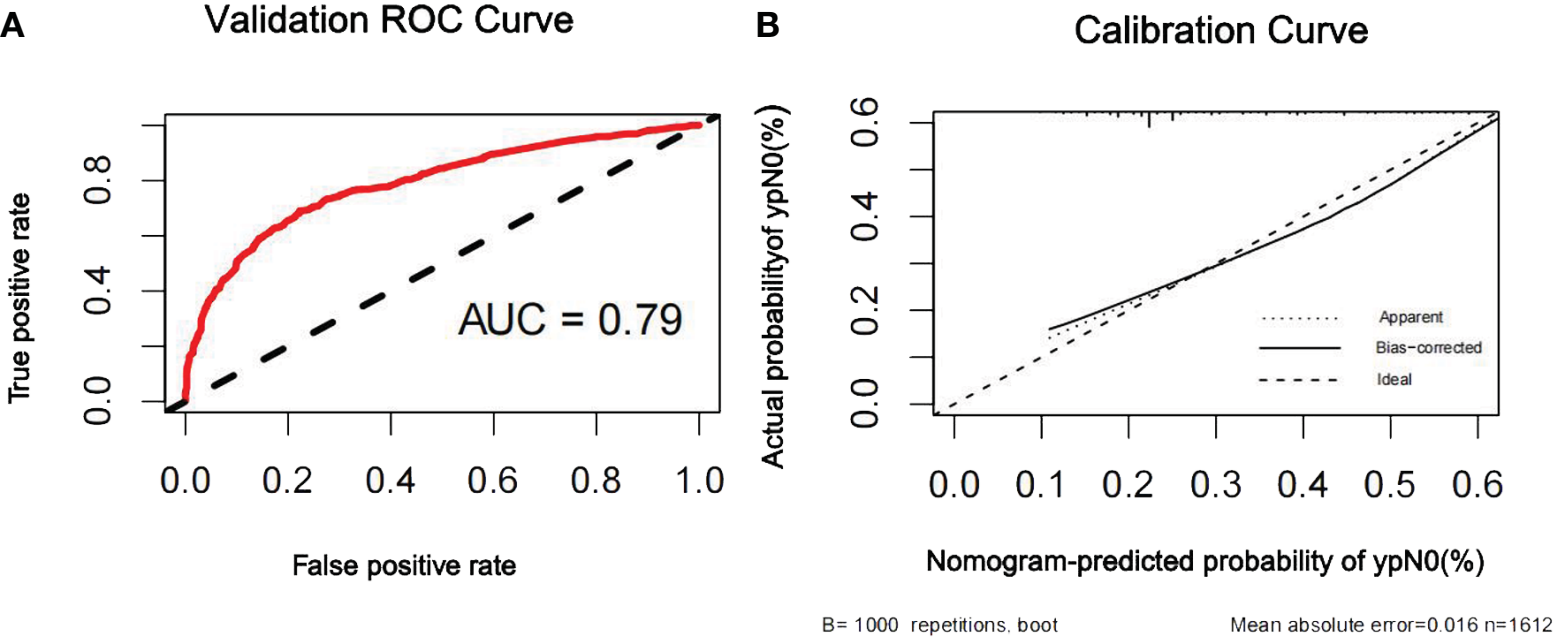
ROC curve **(A)** and calibration curve **(B)** are shown for the prediction model of ypN0 in the internal validation cohort. For discrimination in the internal validation set, the ROC curve indicated an AUC of 0.79. ROC, receiver operating characteristic curve; AUC, area under the curve.

**Figure 5:**
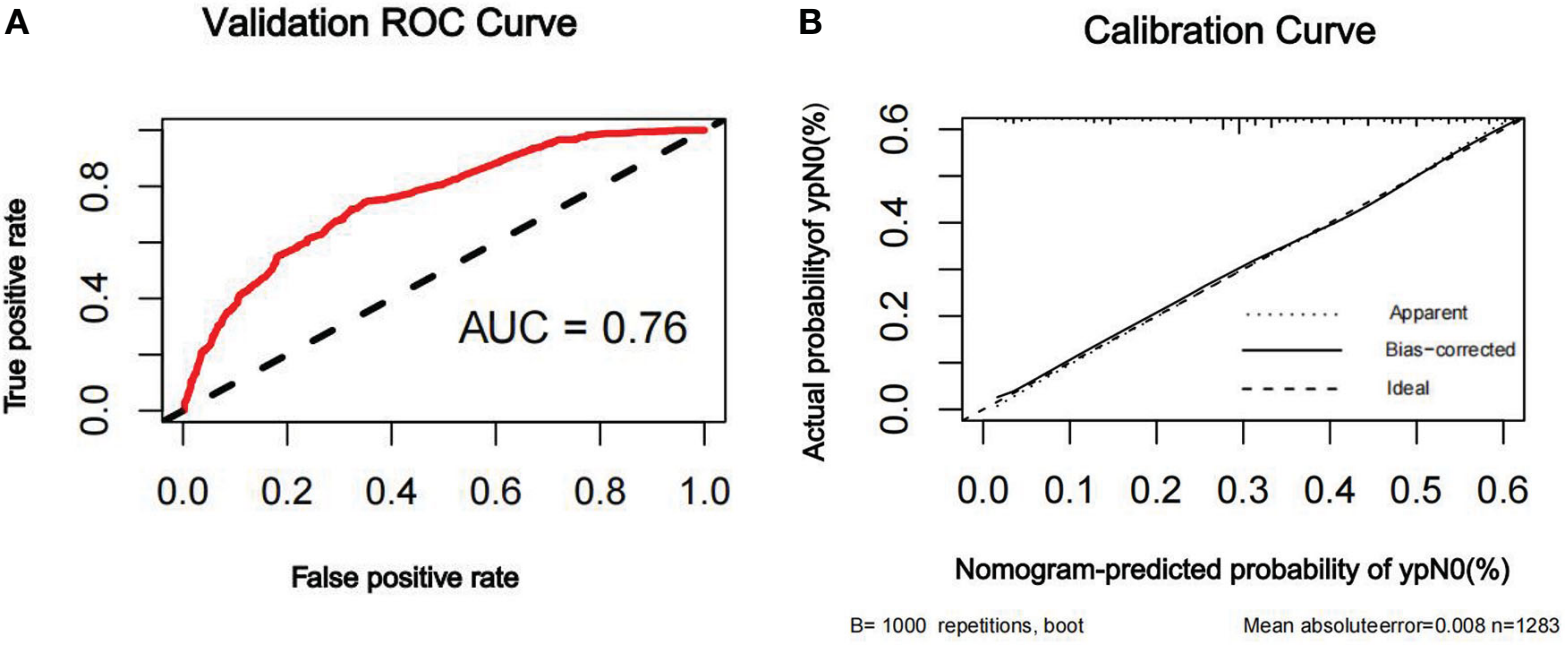
ROC curve **(A)** and calibration curve **(B)** are shown for the prediction model of ypN0 in the external validation cohort. For discrimination in the external validation set, the ROC curve indicated an AUC of 0.76. ROC, receiver operating characteristic curve; AUC, area under the curve.

The discriminatory ability of the nomogram to predict ypN0 status was investigated using receiver operating characteristic (ROC) curve analysis. The AUCs of the training, internal validation, and external validation sets were 0.80, 0.79, and 0.76, respectively, indicating that the nomogram had potentially promising predictive power. The calibration plots presented excellent agreement between the training and validation sets and showed no significant difference between the predicted and actual probabilities of ypN0 (P = 1.000) (**Figures 3**–**5**).

## Discussion

Among patients with cN0 breast cancer, approximately 74% do not have axillary lymph node metastasis (24). This means that even SLNB represents overtreatment and causes unnecessary complications, with few advantages for many patients. However, the St. Gallen Consensus Panel in 2017 (25) and the German AGO recommendation in 2022 (26) recommend SLNB as the standard surgical procedure for patients who present with cN0 before and after NAC. In patients who are cN+ and achieved nodal pCR after NAC, ALND is still performed in clinical practice in some cases because of the unacceptable identification rate and FNR of SLNB (7–9, 27).

Recently, the 5-year survival results of the SOUND trial were published (28). This was a prospective non-inferiority phase III randomized clinical trial that enrolled 1,463 patients with small breast tumors (<2 cm) and a cN0 stage. Patients were randomized in a 1:1 ratio to either the SLNB group or the no axillary surgery group. Interestingly, omission of axillary surgery was not inferior to SLNB in terms of the 5-year DFS and OS. This was a study of patients who underwent upfront surgery. For patients who receive NAC, multiple prospective trials investigating whether axillary surgery can be safely abandoned in selected patients are underway. The European Breast Cancer Research Association of Surgical Trialists (EUBREAST)-01 is a prospective clinical trial in which axillary surgery will be eliminated completely (no SLNB) for initially cN0 patients with radiological complete remission and breast pCR in the lumpectomy specimen (29). The ASICS trial is a non-inferiority, single-arm trial open to both breast-conserving and mastectomy patients in which no SLNB is performed in cN0, triple-negative, or HER2-positive breast cancer patients with a radiological complete response on MRI (30). The results of these trials are expected in to continue for the next 5 years. Meanwhile, the prediction model for axillary nodal burden based on clinical and pathological factors has clinical value and application prospects. In the present study, we presented and validated a model based on nationwide multicenter data of breast cancer patients to predict the possibility of ypN0 disease after NAC. Moreover, to prove its universality, we externally validated the nomogram using patient information from different hospitals.

Researchers at the MD Anderson Cancer Center first proposed that breast pCR is strongly correlated with nodal status after NCT (31). In the present study, 46.2% of patients achieved ypN0 and 20.3% of patients achieved bpCR, and the rate of ypN0 was greater on patients who achieved bpCR than in the nonbpCR group (75.3% vs. 38.7%). Tumor response to NAC was significantly related to tumor subtype. Barron et al. (32) reported 30,821 patients with cT1/cT2 cN0/cN1 breast cancer treated with NAC from the American National Cancer Database and reported breast pCR rates of 37.2%, 58.2%, 37.2%, and 13.1%, respectively. The ypN0 rates were 78.6%, 84.5%, 75.3%, and 47.0% for TNBC, HR−/HER2+, HR+/HER2+, and HR+/HER2− subtypes, respectively. In our study, the distribution of tumor subtypes was consistent with that in the above study; however, the rates of bpCR and ypN0 were low because we included cT3–4 and cN2–3 patients. In addition, the pCR and ypN0 rates of HER2+ patients were not significantly high in the current study, possibly because only approximately half of the patients with HER2+ status (50.5%, 1,335/2,642) received molecular-targeted therapy because the targeted drugs were not covered by medical insurance in the early years.

As expected, clinical nodal stage and breast tumor response strongly predicted ypN0. To avoid the influence of receptors on molecular subtypes, we did not include subtypes in the analysis. We found that patients with ER-negative, PR-negative, and HER2-positive disease had a higher ypN0. Ki67 is a proliferation marker, and patients with higher Ki67 levels showed greater sensitivity to chemotherapy in previous studies (33, 34), which was consistent with our study. In the present study, multivariate analysis revealed that more treatment cycles were associated with ypN0, independent of the tumor histology and treatment regimen. Clinical tumor size has been shown to be a predictor of lymph node status in operable breast cancer patients in several previous studies (35–37); however, in the context of NAC, the relationship between cT and ypN0 was not significant in our study.

We developed a nomogram based on multivariate logistic regression results. In contrast to previous nomograms that predicted axillary pCR in initially cN+ patients (38–40) or in specific subtypes (41, 42), the current nomogram predicted ypN0 in all patients with stage cT1–4N0–3 disease. The AUC of the nomogram in the ROC curve analysis was 0.80, 0.79, and 0.76 in the training, internal, and external validation cohorts, respectively, and showed good discrimination in the prediction of ypN0. The advantage of our prediction nomogram is that most breast cancer patients who receive NAC can be assessed, and the indicators for building the nomogram can be easily acquired by surgeons. Moreover, as mentioned above, our findings are consistent with those of previous studies in a global context in terms of pCR for different subtypes, which indicates that our nomogram can also be applied to patients in different countries.

There are several limitations in our study. First, histological grade was found to be an independent prognostic factor for pCR in patients with breast cancer in previous studies (43, 44). In our study, we could not analyze this factor because it was not included in the initial database. Second, if we put this nomogram into practice for the omission of any axillary surgery, it should be determined before surgery, but bpCR is available after surgery. However, multiple studies have explored methods to detect bpCR without surgery (45–48). A prospective trial showed that image-guided vacuum-assisted core biopsy (VACB) of the primary breast tumor bed following NAC can identify patients who are very likely to have a bpCR with an FNR of <5% (49). Another potential limitation of this study was its retrospective nature. Our study, which reflects the current clinical practices across the country, will facilitate the design of prospective clinical trials in the future.

Future Directions: The developed nomogram may help clinicians weigh the lymph node tumor burden after NAC more appropriately. However, if our research conclusions are extended to clinical work, further clinical trials using this nomogram are required to determine the survival and local recurrence rates of patients who avoid axillary surgery following NAC. The authors expected that related studies of the nomogram could lead to more feasible progress, and that the nomogram could be well connected with targeted axillary dissection, including the clipped node.

## Conclusions

We present a real-world study based on nationwide large sample data and construct a nomogram model that can effectively screen ypN0 to provide better advice for the management of residual axillary disease in breast cancer patients receiving NAC.

## Data availability statement

The original contributions presented in the study are included in the article/supplementary material. Further inquiries can be directed to the corresponding authors.

## Ethics statement

The studies involving humans were approved by Ethical Review Committee of the First Hospital of Jilin University (No. 2021-066). The studies were conducted in accordance with the local legislation and institutional requirements. The ethics committee/institutional review board waived the requirement of written informed consent for participation from the participants or the participants’ legal guardians/next of kin because as this was a retrospective study and all data analyses were performed anonymously.

## Author contributions

AM: Conceptualization, Formal Analysis, Methodology, Software, Validation, Writing – original draft, Investigation. YiJL: Data curation, Formal Analysis, Methodology, Software, Validation, Writing – review & editing. NC: Data curation, Writing – review & editing. ZL: Data curation, Writing – review & editing. RL: Data curation, Writing – review & editing. YZ: Data curation, Writing – review & editing. HY: Data curation, Writing – review & editing. YuJL: Data curation, Writing – review & editing. KL: Data curation, Writing – review & editing. JZ: Data curation, Writing – review & editing. DM: Data curation, Writing – review & editing. ZY: Data curation, Writing – review & editing. YHL: Data curation, Writing – review & editing. PF: Data curation, Writing – review & editing. JW: Data curation, Writing – review & editing. HJ: Data curation, Writing – review & editing. ZZ: Data curation, Writing – review & editing. XT: Data curation, Writing – review & editing. ZC: Data curation, Writing – review & editing. KW: Data curation, Writing – review & editing. AS: Data curation, Writing – review & editing. FJ: Data curation, Writing – review & editing. PW: Formal Analysis, Writing – review & editing. JH: Conceptualization, Project administration, Resources, Supervision, Visualization, Writing – review & editing. ZF: Conceptualization, Data curation, Resources, Supervision, Writing – review & editing. HZ: Conceptualization, Funding acquisition, Investigation, Methodology, Project administration, Supervision, Visualization, Writing – review & editing.
